# Weathering the STORM and Forecasting Equity for Older Black Women: Expanding Social Determinants of Health

**DOI:** 10.3390/ijerph22121777

**Published:** 2025-11-24

**Authors:** H. Shellae Versey, Samuel Van Vleet

**Affiliations:** 1 Department of Psychology, Fordham University, Bronx, NY 10458, USA; 2 Department of Psychiatry, Massachusetts General Hospital, Harvard Medical School, Boston, MA 02114, USA; svanvleet@mgh.harvard.edu

**Keywords:** intersectionality, aging, social determinants of health, stress, retirement, Black women

## Abstract

A strong body of evidence indicates that social determinants impact health. While this research has identified a range of risk factors for health, health equity goals require recalibration further “upstream” towards *structural* drivers of health and aging inequities. Recognizing how systems of power and chronic exposures are embodied and facilitate differential risks and opportunities is important for expanding research at the gender–race–age nexus. Specifically, adopting a structural aging approach can help contextualize health outcomes for older Black women. Drawing from previous research, we explore how structural drivers shape health, examine their impact on Black women’s life experiences, stress exposures, and present a model for interpreting social trajectories of oppression, resistance, and marginalization (i.e., the STORM model) across the lifespan. Extending research on strength, resistance, resilience, and coping may open new opportunities to reframe and understand older Black women’s health. Importantly, developing structural competence can facilitate “seeing structures” and advocating for structural interventions leading to critically minded theory, practice, and policy that properly situate aging processes within broader, intersectional contexts.

## 1. Introduction

Social determinants of health (SDoH) have been recognized as contributors to longevity and psychological well-being, encompassing a broad range of factors and conditions that shape the environments in which people work, grow, and live [1,2]. At the same time, this framework has not been completely integrated with a life course perspective. Growing older unfolds alongside other pressures, processes, and policies (formal and informal) that can systematically exclude groups and individuals, repeatedly and over time [3,4,5]. The intention of this paper is to explicitly link “downstream” risk factors to their “upstream” origins (e.g., systems of exclusion) in an aging context, naming structural power as a root cause. Moreover, a key aim is to connect these relationships to later life outcomes, highlighting processes that undermine older Black women’s health.

Though some of these connections have been made previously [6,7], a structural aging approach is less commonly applied in the gerontological literature, specifically among older, gendered, racialized groups. Before addressing later life disparities and the impacts of ageism, there must be a holistic understanding of the different systems of oppression that evolve and emerge as people grow older. Therefore, rather than framing aging—a natural biological and developmental process—as a risk factor for negative outcomes, or attend to the issue of ageism, which is not equally experienced across all older adults, we explore how aging amid a range of social and structural pressures can contribute to intermediate processes that are important for understanding Black women’s health across the lifespan. Specifically, we examine how marginalization and related stress exposures recur and converge in the lives of older Black women through the lens of weathering and resilience, highlighting key examples.

This perspective is intersectional and represents “an advance over earlier models that assume that advantage and disadvantage simply accumulate,” [8] since the constitution of gender, race, and age are regarded as socially constructed categories that vary as a function of one another. Understanding these constructions, access to resources, and how differences in power drive inequities are key aims of this paper.

## 2. Background: A Structural, Intersectional View of Aging

Researchers have called for investigating how gender, ‘race,’ racism, racialization, and other points of oppression shape opportunities for health and aging [8,9,10,11,12,13]. Yet, comprehensive structural and intersectional frameworks of aging remain scarce. Even when researchers acknowledge macro-level factors, interventions and recommendations for further study are typically focused on proximate levers, such as health behaviors, social supports, and coping strategies, leaving broader systems that cause inequity unaddressed [14]. In contrast, a key aim of intersectional scholarship is to reflect on structural forces and how they impact the social locations of the researcher and researched.

One feature of intersectionality is recognizing the role of structural power and how converging systems (upheld by power) configure everyday life [8]. In this way, intersections of oppression and social position shape experiences of aging or a ‘structural view of aging’—contrary to a focus on individual-level and behavioral factors—describing how “status, resources, and health of, and even the trajectory of the aging process itself, are conditioned by one’s location in the social structure and the relations generated by the economic mode of production…” [15] (p. 19). In other words, the life course is socially constructed and often constructed against those whom the system does not favor. Risks and rewards are unevenly distributed which shapes how individuals reach old age, their experiences in later life, health, and mortality [15,16,17].

## 3. Interrelated Systems with Structural Roots

Interdependent and complementary systems compromise health across multiple levels, and these relationships are best understood from an intersectional perspective that describes how inequality ‘gets under the skin’ via a complex web of causation [1,16,17,18,19]. Similarly to identifying the spider as the architect of the web [1], we suggest that reducing health inequities requires serious attention to root causes (e.g., “fundamental causes”), and how power is embedded in those root causes. In other words, rather than an exclusive focus on health disparities across subgroups of older adults, shifting attention to the spider—systems of exposure that target high-impact levers for change—might be more useful [20,21,22]. Therefore, this paper proposes an expansion to health determinants literature and research on older Black women—a neglected intersection—to better understand less recognized and structural threats to health and aging.

In the following sections, we examine how two forms of structural exclusion, gendered racism and classism, unfold across the life course. To ground this analysis in concrete examples, we focus on processes that significantly impact older Black women—the feminization of poverty, retirement, and care provision (linked to gendered racism and classism), and medical racism—to examine how resource scarcity and chronic (e.g., toxic) stress is embodied and likely expressed in later life. These mechanisms shape health outcomes via employment opportunities, wealth and pay gaps that disadvantage Black women, social norms and socializations, and other factors that give rise to uneven exposures to institutional and interpersonal discrimination and stressors. Finally, we present a conceptual model (i.e., the STORM model) to capture how structural determinants of health derive from a fundamental root cause (e.g., power differences) and are not singular, point-in-time events, but are recursive and ongoing across the lifespan, including waves of resistance and marginalization.

The goal of this paper is twofold; first, we suggest (as have scholars previously) that researchers interested in characterizing and describing health effects within a diverse, aging population should consider the interplay between structural systems of exclusion, marginalization, and differential outcomes across the life course [8,16]. Although this is not a novel suggestion, engagement with structural drivers of inequity (such as power and racism) has been slow. Research on ageism, for example, can benefit from recognizing how age-based exclusion in later life often occurs alongside other forms of cumulative advantage and disadvantage. This broader perspective highlights that ageism is not only interpersonal, but also structural and represents one form of discrimination among many intersecting systems of inequality. Secondly, we argue that scholars interested in researching Black women’s health should consider how threats across the life course shape Black women’s experiences in older age.

## 4. Age, Work, Retirement: Modern-Day Implications of Historical Marginalization

Racialized health inequities (or health disparities) are by-products of historic race relations, as well as current racial hierarchies [9,10,23,24,25]. Life course perspectives aimed at studying aging have traditionally emphasized the timing of life events (e.g., marriage, retirement); however, exposures to other enduring structures and fundamental causes (e.g., racism, sexism, gendered racism) are necessary to consider the full dynamic of older adult life outcomes. Beyond understanding these effects, it is important to consider commonalities as well as differences across various social locations and recognize the ways that institutions (e.g., work, health care) shape health differentially. In a critical context, it is worth questioning whether traditional age-related milestones characterize all older adults, since some groups are necessarily excluded by focusing on social markers rather than actual lived experiences.

For example, some adults never retire, or they work well past retirement age, despite occupying low-paying and low-status positions [26,27]. In fact, Black and Latiné workers (particularly women) are most likely to reach retirement age with the least wealth, reflecting longstanding histories of gendered racism and structural barriers to employment [27]. It is perhaps unsurprising that Black women are engaged in the labor market for longer periods of time since they earn less and spend more for childcare, housing costs, and have a disproportionate amount of their income allocated to repaying student loan debt, limiting financial resources for savings and retirement [28,29,30,31]. Therefore, research on retirement as a marker of older adulthood may not fully account for workers who remain employed past retirement age for a variety of reasons.

Historical events can also shift employment trajectories that reflect modern-day wage gaps. Most Black older adults alive today were born in the American South during the Jim Crow era, attended segregated schools, and experienced both de jure and de facto separatism in everyday life—all which shape education, employment, and health [22,23,24,32,33]. Moreover, Black women employed in domestic and agricultural jobs were largely denied Social Security benefits when the Social Security Act of 1935 was passed, resulting in lost benefits and longer job tenures [25]. Currently, earnings from certain low-paying domestic jobs may not count toward retirement benefits if the worker earns less than a minimum amount, preventing the accumulation of Social Security credits and impacting overall employment-retirement trends.

## 5. Contemporary Feminization of Poverty Among Black Women

Wealth and wage disparities have contributed to the “feminization of poverty” among Black women, which refers to the disproportionate number of Black women living in poverty, compared to other ethnic-racial and gender groups [34]. Despite gains in recent decades, Black women continue to trail White women and Black men in pay and employment opportunities [35,36]. Lower wages and wealth gaps are true for Black women across socioeconomic strata and regardless of marital status [37,38]. These disparities persist *despite* higher educational attainment for Black women compared to Black men.

For example, Black women earn less than men with the same or less education, receiving lower compensation for the same work [39]. Therefore, while not all Black women live in poverty, Black women earn the least and accumulate fewer financial assets compared to all other groups over the span of a career, regardless of class background [38]. This means that, due to a projected history of lower wages, Black women are likely to reach later life with more debt and fewer resources (e.g., less wealth and retirement savings). Over time, the effect of wage disparities, combined with gendered-racial discrimination in access to education, housing, and health care can lead to *lifetime wealth gaps* that contribute to precarity and health risks in later life. The growth of the (Black) feminization of poverty phenomenon connects lifetime wage inequity, disproportionate debt, and material scarcity to health outcomes, and is important in considering how different forms of inequity compound across the life course and present a restricted range of later life possibilities.

## 6. Informal Caregiving: Work Never Ceases

Work is often a complex endeavor for Black women, including formal (paid) and informal (unpaid) responsibilities. Since Black women may be less prepared to retire, they often continue to work and provide care and/or financial assistance to immediate and extended family [40,41]. Regardless of income level, Black women typically assume unpaid caretaking duties while employed and post-retirement [42,43,44]. In fact, Black women tend to be caregivers to multiple people while still engaged in paid work, often depleting cash savings and straining other (e.g., emotional and psychological) resources [45].

Without consideration of work, caregiving, and employment trends, there is a risk of attributing later-life inequities solely to age, rather than recognizing them as the result of lived experiences. Incorporating data on lifetime income, caregiving responsibilities, and occupational disruptions (such as layoffs, missed promotions, or workplace discrimination) can offer a more accurate understanding of health inequities in later life.

## 7. Gendered Racism in Health Care Across the Life Course

While employment and wage restriction redirect tangible resources that would otherwise improve Black women’s health [39], other types of marginalization contribute to direct and indirect health effects, including medical racism and disparate treatment within health care settings [46,47]. The misdiagnosis of health conditions, overlooked symptoms, and negative patient–provider interactions, are frequently reported by Black women as features of routine care experiences [48,49,50,51]. Research indicates that Black women are subject to poorer medical care and increased exposure to gendered racism in health care settings [52,53,54].

For example, in a study of physical referral practices researchers find that women and Black patients were less likely to receive referrals for cardiac catheterization than men or White patients, respectively [53]. In fact, data revealed no significant differences in rates of referrals between Black men, White men, and White women. Rather, Black women received fewer referrals overall; this effect drove the gender and race main effects that led to the original conclusions [55]. These findings support accounts that Black women report feeling unseen, mistreated, and disregarded by the health care system and medical professionals. Overall, estimates indicate that at least 34% of Black women feel their health concerns are not taken seriously by health care providers [56], with these effects extending beyond childbearing years, possibly leading to distrust and disengagement from health care systems long-term. Even with improved access through Medicare, prior experiences of dismissal and discrimination may lead to underutilization in later life.

Taken together, institutional systems and processes should be accounted for in future aging research. While research recognizes that structures and norms are uneven across groups, understanding the convergence of these factors—applying a structural, intersectional lens to aging—has only recently been considered in influencing adult development and quality of life [15,57,58,59].

## 8. Cumulative Consequences: Forecasting a Perfect STORM

As Black women age, life becomes increasingly complex with factors that can attenuate longevity [60,61,62]. Ignoring these contexts renders an incomplete understanding of how health inequities are sustained [63]. Cross-sectional or point-in-time research (e.g., Black maternal health) presents fragmented health profiles that do not fully represent lifetime lived experiences. One relevant process in the context of Black women’s health and recurring exposures is weathering.

## 9. The Weathering Hypothesis: A Response to Structural Marginalization and Resilience as Scar Tissue

*If you’re Black, working hard and playing by the rules can be part of what kills you.*

[64] (p. 3)

On a structural level, repeated instances of marginalization (e.g., racism, etc.), contribute to premature aging on a cellular level, even if you are educated, employed, and healthy. Racism and stress from navigating discriminatory systems increases chronic toxic stress in the body, which increases vulnerability to illnesses and accelerated aging—a phenomenon known as biological “weathering” [65]. The concept of weathering can be applied to the *psychological* consequences of marginalization and stress as well [60,66,67]. For instance, psychological weathering describes negative responses to different forms of racism and gendered racism, contributing to negative emotional states, depression, distress, and intermediate phenomena (e.g., Superwoman schema or SWS) that link the macro- (i.e., gendered racism) and the micro- (i.e., biological aging) [68]. 

A key to understanding weathering is that it is a *responsive* process to structural marginalization and oppression; whether you attempt to cope or resist, say by “working hard and playing by the rules,” the health outcomes can be the same. Put simply, one cannot escape racism or its effects. Although cultural resiliency (e.g., Superwoman schema [69]) is one strategy used to manage racism [70,71,72,73,74,75,76], resilience is still an adverse event, since it only occurs in response to harm or harmful events. As Suslovic and Lett note:

*Given that resilience is a disproportionate expectation of the marginalized, a more appropriate framing for it in the administration of healthcare is a marker of health inequities; an adverse event in response to structural harm that manifests within the individual. We argue that we should reframe “resilience as treatment” to “resilience as scar tissue…”*

[77] (p. 340)

Superwoman Schema (SWS) is a framework that captures how Black women respond to and manage gendered racism, and is defined by emotional suppression, expressions of strength and capability, intense caring for others, and difficulty accepting assistance or support [69]. While in some cases SWS has been associated with adaptive outcomes, in other instances it has been linked to poorer health [78]. Research suggests that Superwoman Schema and other types of high-effort coping strategies possibly compromise mental and physical health [76,79,80] through “weathering,” leading to the early onset of poorer health, across the lifespan and extended into later life [61,65,81,82,83,84]. Therefore, scar tissue in this context represents a marker of Black women’s lived experiences characterized by embodying strength against all odds, while simultaneously undermining efforts that would provide actual strength and support (e.g., accepting help, admitting overwhelm) [76] (p. 2). The weight of carrying strength and stress symbolizes how Black women are socialized early on to cope by resisting vulnerability, yielding scar tissue that may not fade in older age [85].

Since biological weathering occurs at the cellular level, these effects may be less visible, while psychological weathering (e.g., coping by strength, embodying the SWS, and assuming multiple responsibilities) may be obvious and in some cases, praised or encouraged. These factors can set the stage for poorer later life outcomes [11]. Overall, resources that Black women may have drawn on (e.g., strength, advocacy) to challenge gendered racism may not necessarily counter negative effects, and possibly worsens them [48,85]. This dual narrative of resilience is a hallmark of research on Black women’s health and warrants additional study.

We extend this research to suggest that weathering likely unfolds in specific life contexts—work, providing care, retirement, and health care—representing structural–social–biological processes that inform later-life outcomes. At the same time, we recognize that the effects of weathering and its consequences (e.g., health inequities) will remain intact with the continued maintenance of racialized structural power and other mechanisms that require resilience.

## 10. Towards Aging Equity: Structural Intersectionality Across the Lifespan

Broadly, intersectionality describes how systems and flows of power, privileges, and resources disadvantage and privilege groups and individuals. We also use intersectionality as a working epistemological framework to move beyond “health disparities in aging” research from a categorical and comparative perspective, with the aim to encourage new research questions and ways of thinking about aging and later life, using women racialized as Black as a case example. Centering group nexuses, such as gender and race, can help counter what researchers call *intersectional invisibility*, or the erasure of subgroups, such as Black women, from aging research and from broader social consciousness (i.e., older Black women’s issues are not at the fore of women’s health, aging research, or health disparities agendas) [86]. Rather than reinforcing a hierarchy of discrimination types, this approach recognizes that additional dimensions of exclusion in later life (i.e., ageism) may be uniquely experienced by those whose life trajectories have been shaped by multiple, intersecting forms of oppression.

Articulating which systems and contexts are meaningful shifts the focus of analysis from categories to processes (e.g., racism rather than race) and guides research with questions of power in mind [22,55]. Clearly, no single study can assess all levels and manifestations of inequality; however, we recommend directly naming and assessing the contexts and experiences of inequalities—ranging from structural (e.g., laws and policies) to interpersonal (e.g., experiences of discrimination) to intrapersonal—rather than relying on sociodemographic categories as proxies to advance research questions and designs toward intersectionality [55].

Moreover, examining person-centered accounts may highlight points of omission and erasure in the literature, providing a more accurate depiction of systemic barriers and awareness of how these barriers shape clinical and aging outcomes (e.g., structural competence). For example, directly assessing people’s experiences of heterosexism, racism, sexism, and their fusions provides data on how and when these experiences are distinct, linked, or amplified. Assessing experiences and structural indicators, such as policies and processes, also yields data on how structural SDoH relate to settings, risk factors, and community risks and rewards. Therefore, attending to how power inequalities manifest in the lives of people across various sociodemographic locations, how people respond to and resist inequalities, and how inequalities and resistance relates to outcomes can reveal shared and unique manifestations and provide opportunities to improve structural competency by “seeing power” [87]. Ultimately, advancing intersectionality in aging equity research requires conceptualizing, operationalizing, and analyzing intersectional interconnections and grappling with issues of power, inequality, and domination.

## 11. STORM: A Conceptual Framework for Centering Older Black Women’s Experiences

**Figure 1:**
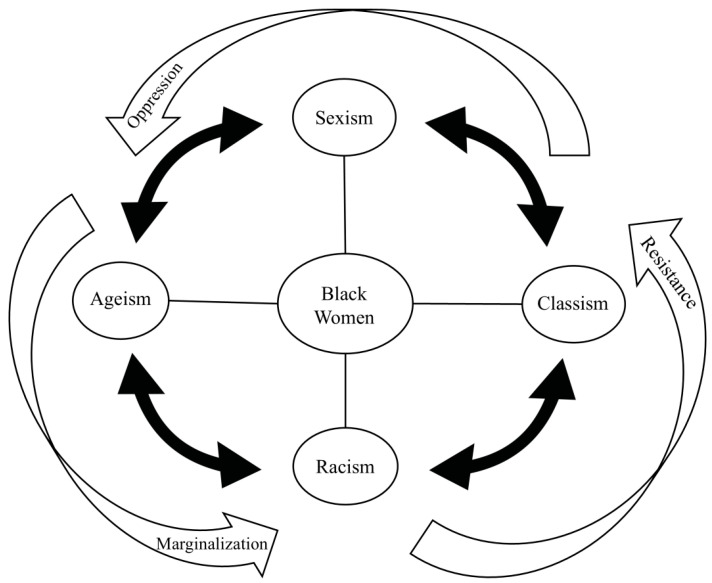
Social Trajectories of Oppression, Resistance and Marginalization (STORM) Framework.

To aid researchers, we propose a conceptual framework representing Social Trajectories of Oppression, Resistance, and Marginalization (STORM model; see Figure 1). STORM reflects interconnections between structural forces, social positions, and lived experiences across the life course. While these interconnections can create turbulence, they can also cultivate strategies of survival, adaptation, resistance, and strength. STORM builds upon cumulative disadvantage and advantage theory [58], intersectionality [88], and weathering [61,83] to localize how systems and contexts shape health. Drawing from previous research, coping strategies used in response to oppression earlier in life can inform coping with other types of oppression later in life [88]. With this understanding, STORM frames the lingering effects of oppression, coping (e.g., resistance), and marginalization repeatedly and across time. This conceptualization draws on elements of prior models, such as the social “web of causation,” positioning Black women as individuals while also placing their experiences within a larger context [89].

Bidirectional relationships represent oppression as being rarely experienced singularly or at one time point, impacting social locations and social mobilities [9,90,91]. STORM provides one conceptualization of upstream exposures and encourages researchers to think structurally about life course determinants of health. Finally, the dynamic relationship between oppression, resistance, and marginalization is shown here as a recursive process, one that is likely to exact “death by a thousand cuts” over a lifetime giving rise to enduring inequities. Taken together, STORM offers a pathway for future research to more fully capture how inequality and resistance unfold across the lives of older Black women.

## 12. Implications and Future Directions

A practical application of the STORM framework begins with building research environments that reflect the diversity and complexity of the populations being studied. This includes the intentional hiring and training of research scholars from diverse backgrounds, conducting research in diverse settings, the use of community-based participatory and critical participatory action research (CPAR), critically reflexive research, critical mixed methods, and the adoption of structural competence and cultural adaptation to encourage researchers to ground approaches and potential solutions in the perspectives of marginalized groups [92,93]. Applying the STORM model can assist researchers in interpreting health and social outcomes of older Black women, as well as accounting for the complexity of what it means to grow older [88,94]. In this way, marginalization is balanced with resistance, where agency and self-preservation emerge within structures of constraint.

**Figure 2:**
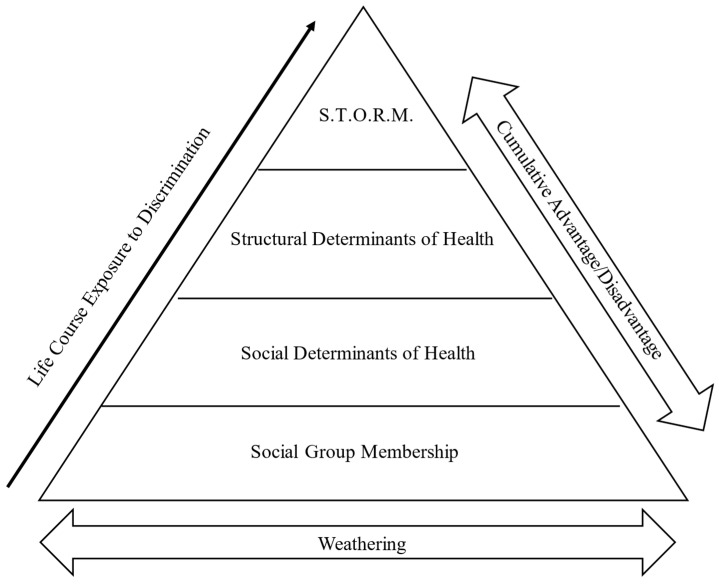
Forecasting Later-Life Inequities.

In conceptualizing how oppression, resistance, and marginalization intersect, STORM should be placed within the context of life course power structures. Figure 2 provides a visual illustration of how cumulative exposure to structural and interpersonal oppression across the life course can influence later life health, socioeconomic outcomes, and psychological well-being in older age. The forecasting diagram (Figure 2) is structured not as a hierarchy, but as a blueprint of the foundational elements of social and structural determinants of health. The bottom layer represents group membership and social location, where over time, different systemic pressures, advantages, and inequities are encountered and navigated. This progression (represented by as the upward arrow on the left side of the diagram) alters life trajectories. Weathering (at the bottom of the diagram) provides one example of the push–pull degradation that occurs as individuals attempt to manage these exposures.

Though cumulative advantages and disadvantages are shown along the right side of the diagram, our model builds upon the initial articulation of accumulation theory (e.g., double jeopardy) to suggest that privileges, risks, and rewards are not simply additive components, but are experienced within a socially constructed realm and dynamic, likely becoming magnified in later life [13,22,95].

In illustrating key points of risk and resistance across the life course, we identify where structural interventions may have the greatest impact and reduce inequity, such as improving access to affordable and quality care, supporting wage equity (e.g., raising the minimum wage and reducing pay gaps), advocating for anti-poverty policies and national paid leave, and eliminating gendered racism in the health care system.

Recognizing structural perspectives enables researchers to anticipate and address barriers that have historically limited participation and representation in gerontological studies. Cultural adaptation and community-engaged approaches strengthen this process by positioning participants and neglected groups (in this case, older Black women) as partners in knowledge production rather than subjects of study. The goal here is not to reinvent existing frameworks but to integrate and extend new ways of knowing by including and amplifying participant voices through collaboration and centering the margins. By doing so, researchers create more equitable and contextually valid interpretations of aging among Black women.

Applying STORM also encourages scholars to carefully investigate assumptions of inherent strength or resilience that often oversimplify Black women’s experiences. Instead, researchers are urged to document how oppression, resistance, and vulnerability coexist within oppressive contexts. Collaborative, person-centered methods and participatory action research can reveal the nuanced ways older Black women navigate overlapping systems of inequality, shedding light on strategies of survival, adaptation, care, and joy that are often invisible in traditional models [63]. The STORM framework guides researchers toward a holistic understanding of aging that acknowledges both structural constraint and personal agency (i.e., an asset-based approach). Future research grounded in this model can inform evidence-based interventions and policies that recognize the interconnected nature of social determinants of health, offering targeted and culturally responsive support to improve the well-being of older Black women.

## 13. Conclusions

As researchers continue to explore later-life inequities, these inequities should be properly contextualized across the life course. The STORM framework provides a lens for understanding how power and lived experiences shape outcomes for older Black women, linking upstream determinants with downstream consequences. This perspective highlights that strategies of survival and resilience often develop in response to systemic challenges, offering insight into the strengths that emerge alongside adversity. While STORM presents older Black women as a case example, the model is intentionally adaptable, as other groups can be similarly located ‘in the eye of the STORM.’ We encourage researchers to refine and extend the framework to examine other populations or contexts, tailoring it to fit specific research questions. By doing so, STORM should initiate a conversation about advancing health equity research, helping scholars design studies, interpret findings, and identify intervention points that respond to structural inequities rather than placing the burden on individuals. Ultimately, STORM offers a flexible and actionable tool to center lived experience, foster equity-driven research, and support interventions that dismantle systemic barriers while amplifying the voices of those most affected.

## Data Availability

No new data were created or analyzed in this study.
